# Challenges and opportunities in adolescent intellectual disability care and rehabilitation

**DOI:** 10.4102/safp.v66i1.5798

**Published:** 2024-02-29

**Authors:** Rakgadi G. Malapela

**Affiliations:** 1 Department of Health Studies, College of Human Sciences, School of Social Sciences, University of South Africa, Pretoria, South Africa

**Keywords:** adolescents, care, challenges, nurse managers, opportunities, rehabilitation service, views

## Abstract

**Background:**

There have been mounting concerns over the lack of proper facilities for adolescents living with intellectual disability (ALWID), and the struggles particularly of the developing world to provide care and rehabilitation services for this population. Care and rehabilitation services are needed to improve this population’s normal functioning but have been scarce or non-existent in most communities.

**Objectives:**

This study aimed to solicit and describe nurse managers’ views of the challenges and opportunities in rendering care and rehabilitation services to ALWID. The study was based on Julian Rappaport’s empowerment theory, which provided a framework for organising essential knowledge while rendering care and rehabilitation services for ALWID.

**Method:**

A qualitative, explorative design was used to solicit nurse managers’ views of the challenges and opportunities in rendering rehabilitation services to ALWID in Tshwane District, Gauteng province, South Africa. Thirteen participants were purposively selected from three rehabilitation centres.

**Results:**

Data were analysed thematically using Braun and Clarke’s six-step method. Five themes emerged relating to challenges and opportunities in rendering rehabilitation services to ALWID. Two themes focussed on challenges, namely inadequate age-appropriate rehabilitation services and a lack of material and non-material resources. Strengthened support systems, partnerships with outside stakeholders, and the promotion of physical activities emerged as opportunities for rendering rehabilitation services to ALWID.

**Conclusion:**

Nurse managers believe rehabilitation services empower ALWID towards autonomy, enhancing their societal function and quality of life with a crucial strong support system.

**Contribution:**

Nurse managers should lead in creating collaboration platforms for ALWID care, promoting partnerships, sharing best practices, and overcoming challenges in treatment and rehabilitation.

## Introduction

Care and rehabilitation services for adolescents living with intellectual disability (ALWID) would be of great benefit to this population especially in developing countries where limited services are available. Despite the United Nations’ Convention on the Rights of Persons with Disabilities, hindrances to its implementation still occur. Such hindrances include: errors, health inequalities and barriers to inadequate healthcare, health-compromising behaviours, poor disease prevention, and health promotion services.^1,2,3^ Moreover, a study conducted in the United Kingdom (UK) found the hospital journey for people with an intellectual disability (ID) and COVID-19 more difficult compared to the general population without ID.^2^ The World Health Organization (WHO) thus called on the World Health Assembly (WHA) Resolution 74.8 to promote the highest standard of health for people with disabilities and achieve Sustainable Development Goal (SDG) 3, Health for all.^3^

Rehabilitation is defined as ‘a goal-oriented process aimed at promoting optimal mental, social, and physical functioning levels to achieve a quality of life’.^4^ In this regard, it is necessary to explore rehabilitation services’ challenges and opportunities to assist ALWID in achieving optimal functioning. In the Republic of South Africa’s *Mental Health Care Act* (No. 17 of 2002),^5^ rehabilitation is viewed as a form of care and service given to individuals to maximise their potential and achieve independent functioning. Based on these definitions, rehabilitation services play an influential role in shaping the lives of ALWID and optimising their functioning. In South Africa, the child and adolescent mental health guidelines advocate for the promotion of all children and adolescents’ development regardless of any mental health problems^6^ and rehabilitation services play an instrumental role in ALWID’s development. Furthermore, these guidelines emphasise general intervention strategies for individuals with genetic or chromosomal abnormalities to reduce the risk of comorbidities and promote the effects of protective factors. This calls upon nurse managers to determine the challenges and opportunities related to ALWID’s rehabilitation services.

It is estimated that one in seven adolescents experience mental disorders,^7^ including IDs. Globally, approximately 166 million adolescents have mental disorders, and of these, 14.9% have an ID. An adolescent is defined as a young person in that transitional phase that occurs between childhood and adulthood, typically spanning from 10 to 19 years of age.^8^ It has also been determined that 30% more males are diagnosed with IDs than females, particularly mild IDs.^9^ Furthermore, one-third (30%) of ID cases are attributed to non-genetic causes, and around 70% are of genetic origin. According to the diagnostic criteria for IDs in the Diagnostic and Statistical Manual of Mental Disorders (DSM-5), the American Psychiatric Association defines ‘ID’ as a deficit in intellectual and adaptive functioning, which is evident during childhood or adolescence.^10^ However, Ginis et al.^1^ indicate that disability should be regarded as a continuum of the human condition and not a condition to be treated with medication. In a systematic review by Nevala et al.,^4^ ID is defined as a significantly below-average intellectual ability to understand and learn new and complex skills (impaired intelligence), and cope independently (restricted social functioning). In this study, an ALWID is aged between 10 years and 21 years and experiencing limitations in intellectual, social, and adaptive functioning. Challenges and opportunities in rehabilitation services should thus be explored to determine positive factors promoting ALWID’s independence.

Extant literature mentions that ALWID face challenges in accessing suitable health services and are prone to more physical illnesses, health inequalities, and limitations when compared to the general population.^1^ The need for further research to better the lives of ALWID has also been reported.

### Problem statement

Literature has recommended interventions that support rehabilitation services to enable the optimal functioning of individuals with IDs.^4^ Lakhan^11^ asserts that rehabilitation cannot be achieved in a single institution; a community-based approach to rehabilitation is required. Intellectual disability is a permanent condition associated with high morbidity and care costs that requires long-term support.^9^ Therefore, it is important to explore and address challenges associated with rehabilitation services to reduce the negative impact on individuals with IDs, their families, health systems, and the state. This phenomenon prompted the researcher to conduct this study to explore nurse managers’ views of existing challenges and opportunities in rendering rehabilitation services for ALWID. The researcher asked: ‘What kind of support is needed to address the challenges nurse managers face while rendering rehabilitation services for inpatient ALWID in Tshwane District, Gauteng Province of South Africa?’ This population’s rehabilitation requires further attention; hence, this study was undertaken to solicit and describe nurse managers’ views of the challenges and opportunities to rendering care and rehabilitation services to ALWID.

### Rappaport’s empowerment theory

Empowerment is defined as ‘a process or a mechanism in which people, organizations, and communities gain mastery over their affairs’.^12^ Freire^13^ posits that Rappaport’s empowerment theory – applied to health – should be understood as an enabling outcome. This notion was supported by Anderson and Funnel,^14^ who claimed that empowerment is viewed as an outcome if it enhances an individual’s independence. In the context of this article, empowerment refers to enablers that can help ALWID become more autonomous. It further maximises their independence and improves their health and wellness.^15^

The empowerment theory was relevant to determine available opportunities for a conducive rehabilitation service. Empowerment entails interactive processes between the individual and the conducive environment.^16^ The primary focus of an empowerment theory looks at obstacles that prevent empowerment and rehabilitation for ALWID. By exploring nurse managers’ challenges and opportunities in rendering services, information could be obtained that enables ALWID to live empowered lives. The purpose of this study was thus to develop insights into nurse managers’ challenges and opportunities in rendering rehabilitation services to ALWID to improve their quality of life. The objective was to explore these challenges and opportunities and recommend supportive measures to improve rehabilitation services for ALWID.

## Research methods and design

### Research design

An exploratory, descriptive design was undertaken to obtain multiple views from nurse managers about their challenges and opportunities in rendering rehabilitation services for ALWID. A non-probability purposive sampling method was used to select nurse managers who were experienced in rendering care, treatment, and rehabilitation services for ALWID.

### Study population

Nurse managers were recruited from three institutions that admit ALWID from 10 years to 21 years. This population was selected to solicit and describe nurse managers’ views of the challenges and opportunities in rendering care and rehabilitation services to ALWID. The recruitment period was from May 2022 to June 2022.

### Inclusion criteria

Nurse managers directly involved in providing care, treatment and rehabilitation to ALWID and who were willing to voluntarily participate in the study were included. Those who gave written informed consent to participate in the study and had more than 2 years’ experience, irrespective of race, mixed race, and religion, participated in this study. Nurse managers had to be working in institutions in the Tshwane district of Gauteng that admitted ALWID to be included.

### Sampling method

A purposive nonprobability sampling method was followed to select eligible participants from the three institutions until data saturation was reached. These participants were selected to achieve the study’s purpose and respond to the research question.

### Sample size and settings

A total of 13 nurse managers participated in the study; 15 individuals were initially invited, but 2 declined to take part. Data saturation was reached at participant number eight, but the researcher conducted five additional interviews to ensure no information that might contribute to the study’s findings was omitted.

The sampled nurse managers were at the forefront of ALWID’s care, treatment, and rehabilitation. They were recruited from three public and non-governmental institutions that render inpatient care, treatment, and rehabilitation services for ALWID in the Tshwane district, Gauteng province of South Africa. These care and rehabilitation centres were deemed fit for this study as they cater for inpatient care and rehabilitation services for persons aged between 10 years and 21 years with severe and profound ID. The average length of a stay is between 40 days and 120 days. These institutions were purposively selected as they serve as referral centres for other provinces such as Gauteng, Limpopo, Mpumalanga, and North West. The recruitment period was from May 2022 to June 2022.

### Data collection

Data were collected from June 2022 to August 2022, using individual, semi-structured, face-to-face or telephonic interviews. Interviews (Online Appendix 1) were conducted in English, lasted 30 min to 60 min, and were audio recorded to guarantee no information was missed. An interview guide was used to maintain consistency during the data collection process.

### Pilot testing the data collection instrument

To uncover problems and avoid ambiguity, the interview guide was tested with three participants. No flaws in the interview guide were discovered, but the results of this pilot testing were not incorporated into the research findings even if they stayed the same. Pilot testing was conducted prior to the actual data collection to evaluate the effectiveness of the instrument, ensuring that it was appropriately designed to gather the desired data.

### Data analysis

The data were analysed guided by Braun and Clarke’s^17^ thematic analysis process following these six steps: (1) familiarisation with the data, (2) generating initial codes, (3) searching for themes, (4) reviewing the themes, (5) defining and naming the themes, (6) producing the final report. Interviews were transcribed verbatim, and the transcripts were read several times so that the researcher could become familiar with and immersed in the data to arrive at the findings. An independent coder not involved in this study assisted in data analysis to ensure objectivity. Data were coded and categorised into five themes: inadequate age-appropriate rehabilitation services; lack of material and non-material resources; support systems; partnerships with outside stakeholders; and the promotion of physical activities.

### Ethical considerations

Ethical clearance for the study was obtained from the University College of Human Sciences Research Ethics Committee with CREC Reference number: 90428595-CREC-CHS-2022 and NHREC Registration number: Rec. 240816-052. Ethics approval was granted from 22 April 2022 to 22 April 2025. This study followed the Declaration of Helsinki (2013). No patients were involved in the study. Participation was voluntary, and informed consent was obtained from nurse managers directly involved in the care, treatment, and rehabilitation of ALWID. Participants’ freedom to withdraw from the study at any time was upheld; two participants declined without prejudice. Pseudonyms were used to protect the participants’ identities, and data were collected from June 2022 to August 2022 at the participants’ convenience to minimise disruptions. Information collected from the participants was kept confidential and private in a lockable cupboard in the researcher’s office. Documents saved to the researcher’s laptop were password encrypted to prevent unauthorised individuals from gaining access to the data. Participants were reassured that confidentiality and anonymity would be maintained throughout the research process.

## Results

Thirteen participants (nine females and four males) were involved in this study and most participants were females. Their ages ranged from 42 years to 64 years, their work experience was between 11 years and 34 years, and most had relevant qualifications; the highest being a master’s in psychiatric nursing science. These demographics (see Table 1) illustrate that participants were eligible to participate in the study.

**TABLE 1:** Demographic profile of the participants (*N* = 13).

Participants’ demographics	Frequency	Percentage
**Age group**		
40–49 years	3	23
50–59 years	9	69
60–69 years	1	8
**Gender**		
Female	9	69
Male	4	31
**Highest qualifications**		
Diploma	3	23
Post Graduate Diploma	1	8
BCur Degree	5	38
Masters	4	31
**Area of employment**		
Public	12	92
Non-governmental Organisation	1	8
**Years of nursing experience**		
10–19 years	6	46
20–29 years	5	38
30–39 years	2	16
**Rank**		
Operational manager	12	92
Deputy director	1	8

Inadequate rehabilitation services and a lack of material and non-material resources emerged as primary challenges. Conversely, three opportunities emerged, namely strengthened support systems, partnership with outside stakeholders, and the promotion of physical activities. These play an influential role in providing effective rehabilitation services (refer to Table 2 for the emerging themes).

**TABLE 2:** Overview of emerged themes from the study.

Category	Themes	Sub-themes
Challenges	Inadequate age-appropriate rehabilitation services	-
	Lack of material and non-material resources	-
Opportunities	Strengthening support systems	Family support structural support multidisciplinary team support
	Partnership with outside stakeholders	
	Promotion of physical activities	

### Inadequate age-appropriate rehabilitation services

Most participants reported various challenges impacting care, treatment, and rehabilitation services being offered at their facilities. This makes it difficult to empower ALWID to achieve positive outcomes, as indicated in the following statements:

‘The environment does not allow them to be teenagers. Certain activities are not done to stimulate the teenager inside of them that we don’t even know. Currently, we treat them by their mental age instead of their chronological age, if we can merge the two, they will thrive.’ (Sarah, 42-year-old female, telephonic interview)

‘Mental health is now lacking as far as care and rehabilitation services are concerned, so we need to create a conducive environment.’ (Pitbull, 50-year-old male, face-to-face interview)

‘We should not trigger frustrations in our patients by treating them like adults because of how they look while forgetting their mental disability. They need to be involved in sports activities, indoor games and to be taken out regularly for entertainment to keep them busy and not trigger their emotions.’ (Rhee, 52-year-old male, face-to-face interview)

### A lack of material and non-material resources

Most participants emphasised a lack of material and non-material resources when rendering rehabilitation services. This view is reflected in the following statements:

‘The challenge we face is we don’t get resources so it’s difficult for us to work. In terms of monetary, and structural including human resources as well and makes it difficult to work.’ (Sarah, 42-year-old female, telephonic interview)

‘An unconducive environment can create patients to be hostile and irritable, but you need to find out what it is that they need exactly, Situational analysis will indicate whether patients need a closed environment or a more open one where they can be able to play. Another thing is that they will need proper nutrition, if not well taken care of, patients can develop irritations’ (Coloured, 47-year-old female, telephonic interview)

‘We don’t get to be financed well in our institutions; our budgets are always cut. We are short of work staff.’ (Peter, 54-year-old male, telephonic interview)

‘If you do not have the right staff the place will be dirty, and children will be sick.’ (Johanna, 50-year-old female, face-to-face interview)

### Strengthened support systems

Participants indicated that families offered support to ALWID. Furthermore, participants called for structural support to better the lives of ALWID. Under strengthened support systems, three sub-themes emerged, namely family, structural, and multidisciplinary team support.

#### Family support

The following statements emphasise the need for family support:

‘Encourage family support, which is the most important thing because we all have our social networks/families where we come from despite our disabilities. Having parents and team to provide sufficient support is a booster’ (Thando, 53-year-old female, telephonic interview)

‘The involvement of family is the most important thing ever; families are not visiting the patients.’ (Coloured, 47-year-old female, telephonic interview)

‘The inclusion of a social worker to assist in terms of keeping ties with family so that our patients don’t get depressed if they don’t see their family members’ (Rhee, 52-year-old male, face-to-face interview)

‘Their families are unable to take care of them because intellectually disabled patients are fully dependent on environmental care. They have bipolar and depression especially patients that don’t receive support from their families. When they’re depressed, they are throwing tantrums and harm their physical self.’ (Rhee, 52-year-old male, face-to-face interview)

#### Structural support

Quotes in support of the need for structural support are as follows:

‘They are like children, so the setup and environment must be appealing like a creche and colourful with play areas. The structure should fit their intellectual capacity. We need to have more extra mural activities, toys, games, and physical activities. Infrastructure should be appropriate for the mental level of our patients. Constant renovation and change are needed because they are destructive.’ (Rhee, 52-year-old male, face-to-face interview)

‘The structure needs to be user-friendly. The structure must have all the necessary things such as a courtyard and a hazard-free environment.’ (Pitbull, 50-year-old male, face-to-face interview)

‘The structure in its design must be safe for mentally disabled adolescents because the patients can easily use anything to harm themselves because of their mental limitations. The structure needs to be modified to suit the patient’s needs.’ (Didi, 53-year-old female, telephonic interview)

‘A healthy environment will minimise injuries and accommodate their hyperactivity with enough space to room around. A healthy environment will decrease the patients’ and nurses’ stress levels.’ (Peter, 54-year-old male, telephonic interview)

#### Multidisciplinary team support

Twelve participants emphasised the need for a multidisciplinary team to render comprehensive rehabilitation services. The nurse managers also mentioned some opportunities to improve rehabilitation services, stated as follows:

‘Include other multidisciplinary team members to help in assessing and evaluating them. The inclusion of a social worker and speech-therapy also play a role in rehabilitation. Podiatrists are also needed because our patients like to walk barefoot.’ (Rhee, 52-year-old male, face-to-face interview)

‘If we have enough occupational therapists to help with the users for their stimulation that will be a good thing. Qualified dietitian is also important for our users who will understand the type of food our users need.’ (Sarah, 42-year-old female, telephonic interview)

‘We need a multidisciplinary team, which is doctors, nurses, occupational therapists, physiotherapists, and assistants that will help these children to be happy and to take full care of them’ (Johanna, 50-year-old female, face-to-face interview)

### Partnership with outside stakeholders

Participants mentioned several aspects that influence opportunities for partnerships with outside stakeholders:

‘We also need to build a rapport to promote a trusting relationship with our patients and all stakeholders.’ (Betty, 57-year-old female, face-to-face interview)

‘Partnership with NGOs [*non-government organisations*] like Revlon for their make up to boast their self-esteem.’ (Tannie, 64-year-old female, face-to-face interview)

‘They interact with others outside people. Without playgrounds, the children will stay in their rooms and there won’t be any training leading them to sit in wheelchairs unfit.’ (Johanna, 50-year-old female, face-to-face interview)

### Promotion of physical activities

Participants reported the need for physical activities, as mentioned in the following quotes:

‘Without playgrounds, the children will stay in their rooms and there won’t be any training leading them to sit in wheelchairs unfit.’ (Johanna, 50-year-old-female, face-to-face interview)

‘We need toys, equipment and a hazard-free courtyard and equipment in their care to keep them active and busy.’ (Pitbull, a 50-year-old male, face-to-face interview)

‘We need to stimulate them accordingly by providing activities that merge their mental and chronological age. Providing playing games, and music classes to stimulate their abilities. If we can have enough occupational therapists to help with the users for their stimulation that will be a good thing’ (Sarah, 42-year-old female, face-to-face interview)

‘A situational analysis is to be conducted to determine the type of environment needed whether close or open to performing play activities and tasks for intellectually disabled adolescents.’ (Coloured, 47-year-old female, telephonic interview)

‘Considering their psychomotor and mobility problems, they need appropriate activities that will reduce their hyperactivity. Toys for activities and a conducive environment to allow them to roam around. Safety is important because of muscle weakness’ (Peter, 54-year-old male, telephonic interview)

‘I think we need activities that we can occupy users with. For now, our users like music, balls, and colouring.’ (RK, 51-year-old female, telephonic interview)

## Discussion

The study’s findings revealed that nurse managers encountered various challenges while rendering rehabilitation services to ALWID. They particularly mentioned limited resources making it difficult to provide appropriate rehabilitation services. According to Ginis et al.,^1^ individuals living with disabilities encounter greater challenges and health inequalities than those without disabilities. Limited access to rehabilitation and other healthcare services will have a detrimental effect on improving these individuals’ levels of functioning.^9^ Therefore, Hankle et al.^18^ asserted that individuals with IDs will benefit from activities that offer fun and entertainment. Music, sports, and video games could promote opportunities for these individuals to interact with families, friends, and the community.^19,20,21^ In addition, Nyman and Teten^22^ pronounced that video games should be played at the end of the day as they are beneficial to ALWIDs’ quality of life and improve their physical activity. This view was supported in other studies^23,24^ that indicated that active video games are gaining popularity and are essential for stimulating cognitive skills. Stimulation is required to merge the ALWID’s chronical and mental age; this is necessary to stimulate the unknown self, which may lead to self-discovery. The process may assist in identifying the adolescents’ weaknesses and strengths.

Most participants advocated for the need for playgrounds, music, and toys, and believed this would empower ALWID to deal with their frustrations and keep them busy. Similarly, Lancioni et al.^25^ suggested video games and exercise to encourage ALWIDs engagement in physical activity using stimulation-regulating technologies. However, the study does argue that the use of video games may not be suitable for participants with extensive motor impairments.^25^ Increased physical activity has also been found to reduce comorbidities and obesity, and promote health and fitness.^26^ A total of 100 min - 150 min per week performed over a minimum of 2 days is recommended for these adolescents to live a long, healthy life.^27,28^

Creativity and innovation are required to make physical activities more engaging and pleasurable.^29^ This means adolescents’ tastes and preferences should be considered to stimulate active involvement and instil a sense of happiness. According to a study conducted in Sweden, an increase in sports and general activities is beneficial, along with assistance from families, teachers, and the community.^30^ Previous research suggested using cyclic therapies to increase cognitive skills and physical activity.^31^ The ALWIDs are clearly suited to physical activities, which would improve their quality of life.

To maximise their potential and opportunities, they need access to medical, behavioural, and psychological interventions on a community or day-care basis.^9^ This implies that rehabilitation centres need to partner and engage with other neighbouring community-based rehabilitation partners to address limited services. This view was shared by the participants, who mentioned that partnerships with other stakeholders would be beneficial. Furthermore, support from parents, families and the multidisciplinary team will add more value and ensure these adolescents are cared for in totality.

According to Kishore et al.,^9^ family and parental support are critical to providing long-term care for ALWID. Family assessments should be conducted to determine their needs, coping strategies, and support structures to deal with the challenges related to caring for ALWID. Furthermore, Nevala et al.^5^ concluded that family support would enable ALWID to participate in society and obtain better employment opportunities.

Structural support with enough resources to promote a conducive environment also plays a major role in this population’s care. Lakhan^11^ stated that a community-based approach is necessary to enable individuals with IDs, their parents, and the community to promote patient rehabilitation and inclusion. Kishore et al.^9^ also mentioned that settings and structures should encourage a play-based approach and culturally rooted practices that are available and appropriate to the individual’s and family’s needs. Introducing practices that are not tailor-made for adolescents will overwhelm both the individuals and families, and will hinder the rehabilitation process. A multidisciplinary approach is thus needed to address the holistic needs of ALWID throughout their lifespan.^8^ In addition, they need education, prevocational training, and independent living skills related to independent mobility, physical care, communication, modified curricula, aids, occupational and vocational activities, and medication to treat comorbid medical conditions.

Therapists and other professionals also assist with communication difficulties and familial responses to make decisions in everyday situations.^32^ Therefore, the multidisciplinary team’s involvement is needed to address the complex needs of ALWID and enable these adolescents to achieve positive outcomes and live independently. Hahn et al.^33^ recommended that nurse managers connect with team members on patient care needs and have a sense of purpose to find joy in their work and balance their roles. As a result, nurse managers will be able to effect change and offer support in the field of rehabilitation services.

### Limitations of this study

The study was limited to nurse managers directly involved with ALWID admitted in three selected rehabilitation centres in Gauteng province, South Africa. To obtain multiple perspectives, other multidisciplinary team members could have been involved. Therefore, the study’s findings cannot be generalised and used in other contexts.

### Implications for nursing management

Nurse managers should be at the forefront of creating change when rendering care, treatment, and rehabilitation services for ALWID. Nurse managers should advocate for ALWID to receive quality patient care. Families and communities should also be empowered to actively participate in these care and rehabilitation services. Nurse managers must ensure that physical activities and stimulation are consistently provided to empower ALWID to achieve independent living and promote their physical well-being. The use of technological stimulation systems should be encouraged to address the limited resources available in rehabilitation services.

## Conclusion

This study solicited nurse managers’ views of the challenges and opportunities they encountered when rendering rehabilitation services for ALWID. Several challenges in rehabilitation services were attributed to a limited budget, which has led to a lack of resources and poor staffing. Nurse managers also reported some opportunities in terms of stakeholder partnerships. They agreed that appropriate rehabilitation services would empower ALWID to live more independently. The results of the study provide evidence in favour of the empowerment theory, which emphasises that enabling positive outcomes can be attained by providing ALWID with improved opportunities that enhance their independence and overall quality of life. Future studies from the perspectives of patients and carers should be pursued on some level to improve services.
